# The Olympics and harm reduction?

**DOI:** 10.1186/1477-7517-9-33

**Published:** 2012-07-13

**Authors:** Bengt Kayser, Barbara Broers

**Affiliations:** 1 Institute of movement sciences and sports medicine, University of Geneva, 10, rue du Conseil Général, 1205, Geneva, Switzerland; 2 Unit for Dependency in Primary Care at the Department of Community Health and Primary Care, University Hospitals of Geneva, Geneva, Switzerland

## Abstract

The current anti-doping policy (‘war on doping’) resembles the ‘war on drugs’ in several aspects, including a zero-tolerance approach, ideology encroaching on human rights and public health principles, high cost using public money for repression and control, and attempts to shape internationally harmonized legal frameworks to attain its aim. Furthermore, even if for different reasons, both wars seem not to be able to attain their objectives, and possibly lead to more harm to society than they can prevent.

The Olympic buzz is mounting and we can expect multiple headlines in the media on doping and anti-doping stories related to this event. In this article we describe current anti-doping policy, reflect on its multiple unplanned consequences, and end with a discussion, if lessons learned from harm reduction experiences in the illicit drugs field could be applied to anti-doping.

## Editorial

**Figure 1:**
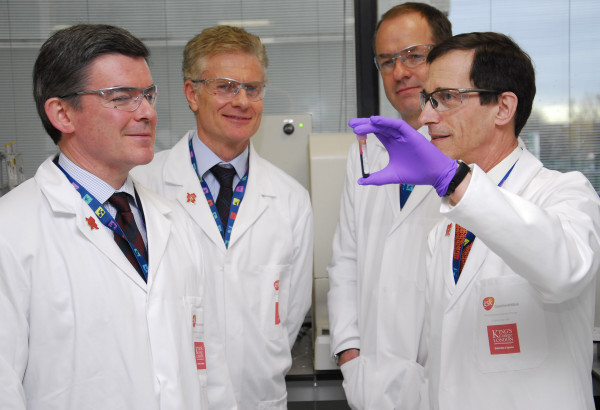
From right to left: Prof David Cowan from King’s College London’s Drug Control Centre and Director of the London Anti-Doping Laboratory, Sir Andrew Witty, CEO of multinational GSK (laboratory sponsor), Paul Deighton, London 2012 Chief Executive, and Hugh Robertson, Minister for Sports and the Olympics (LOCOG, with permission).

Early this year the unveiling of the brand new 2012 London Olympics anti-doping lab made the headlines worldwide, accompanied by strong anti-doping messages for prospective Olympians and illustrated by an iconic photograph depicting the laboratory’s head showing a blood sample to the Minister for Sports and the Olympics (Figure 1). The 4’400 m^2^ laboratory, sponsored by a multinational pharmaceutical company, will be operating 24 h/day during the Games, analysing urine and blood samples of one out of two participating athletes while a part of the samples will be stored for eight years, using the threat of future testing technology as a further deterrent. With the Olympic buzz mounting and the Games monopolizing the media, the moment seems right for debate on drugs and sports among those concerned by drug policymaking and the harm reduction movement in specific. But you may ask yourself: What have the Olympic Games to do with harm reduction? More than you might think.

### The world anti-doping agency

The Olympic movement was the driving force behind the anti-doping efforts that led to the establishment of the World Anti-Doping Agency in 1999. A series of widely publicized doping scandals and public outrage in the nineties triggered this increasingly strong movement advocating doping-free sports. WADA aims at eradicating doping by harmonizing anti-doping practices worldwide from a zero-tolerance standpoint. It is backed by the UNESCO anti-doping convention, now signed by a majority of UN member states. These anti-doping efforts were recently dubbed ‘war on doping’ [1], echoing the declaration of the ‘war on drugs’ by president Nixon in 1971. These wars on drugs and on doping share various features, such as similarities between policies based on zero-tolerance, repression and surveillance, black markets controlled by organized crime, attempts to shape internationally harmonized legal frameworks, ideology and political convenience anchored in media-fuelled moral outrages. Furthermore, when WADA was established and drafted its first list of forbidden substances, the question rose on whether cannabis derivatives should be on the list. They were included largely because of pressure from the ‘war on drugs’ movement, even though there are no known proven performance enhancing effects but rather evidence for the contrary [2,3]. Even though only forbidden during competitions, regularly athletes are indicted because of urine traces of cannabis metabolites, which may remain measurable up to weeks after use. Cocaine metabolites are also regularly found and the public announcement of such cases often leads to important media coverage, strongly condemning the athlete, even if the substance was taken in a recreational context and not for sport performance enhancement. Whereas the ‘war on drugs’ proved to be a failure and public debate is now slowly shifting towards finding better policies - based on public health principles - to deal with psychotropic drug use in society, in sport current policy is still essentially based on repression and surveillance from a zero-tolerance viewpoint. As such anti-doping thus provides the ‘war on drugs’ movement a convenient backdoor to continue advocating its zero-tolerance ideology.

### What is doping?

The readers of this journal are perhaps not fully aware of what is considered as doping and how anti-doping works. Operationally, in sports, doping is defined in the ‘*Code’*[4]: “*the occurrence of one or more of the anti-doping rule violations set forth in Article 2.1 through Article 2.8 of the Code*”. Violations of the anti-doping rule include not only the use or attempted use of prohibited substances, but also the presence of a prohibited substance, or its metabolites or markers, in an athlete’s urine or blood sample; violation of the athlete’s obligation to inform about his/her ‘whereabouts’ (see below); tampering or attempted tampering with doping control procedures; possession of prohibited substances or the means for performing prohibited methods; and trafficking or attempted trafficking in a prohibited substance or the means for performing a prohibited method [4]. Important is also the so-called ‘strict liability’ rule, which states that an athlete is responsible for the presence of a substance or its metabolites in a bodily specimen, whatever the way it was introduced into the body.

### The complexity of anti-doping

Upon first glance the anti-doping rule may seem reasonable and simple, but when looking more closely at the consequences of the rule, questions arise. Some recent examples of indictments for doping, illustrating the complexity of anti-doping policy implementation, are given hereunder.

Dwain Chambers, a famous British 100 m runner, tested positive for THG (tetrahydrogestrinone) in 2003 and was banned from competition for 2 years. The British Olympic Association, on the basis of a bylaw, excluded him for life from participation to the Olympics. The Court of Arbitration for Sports (CAS) overruled this decision in April 2012 after an appeal by WADA, opening the way for Chambers to compete in this summer’s Olympics. The case is illustrative for several reasons. First because of the substance THG (aka ‘*The Clear’*). It was designed by a clandestine laboratory specifically for doping purposes, and by consequence unknown to the scientific community and anti-doping laboratories in particular. It was discovered when a coach sent a syringe containing traces of the substance to the USA anti-doping agency. Second because there are no published experimental data confirming the alleged performance enhancing effects of the substance. Third, since WADA convinced CAS to overturn the ruling of BOA on the basis of non-compliance to universally applicable WADA rules. And fourth, because of the strong condemning of this CAS ruling by many prominent members of the British sports-establishment which seemed to indicate that a doping offence is not seen in the same way as most other offenses in society; if in general, upon punishment for transgression of a rule, one is offered a second chance, a doping offense is deemed essentially unforgivable and worth exclusion of sports for life. As Sebastian Coe, chairman of the London Organising Committee for the Olympic Games, said: *“I am clear cut on the Chambers case – I don’t think there is room for drugs cheats in sport”.*^a^

Alberto Contador, a famous Spanish road-cyclist and Tour-de-France winner, was indicted in 2012 for trace levels of clenbuterol in urine samples obtained during the 2010 Tour. Several aspects set this case apart. First because of the long time it took to condemn Contador, contrasting his case with several other similar cases in other, perhaps less famous athletes. Second, the traces found were so low as to exclude any significant physiological performance enhancing effects around the time of sampling. Third, the excuse used by the defence: ingestion of contaminated meat. And fourth, the widely publicized rumours about possible blood doping as the source of the substance, by transfusion of Contador’s own blood, extracted and stored at an earlier time when doping, on the basis of traces of plastic residues in his blood, compatible with the use of blood bags and tubing, even though his blood parameters did not indicate blood doping.

Christine Ohuruogu is a very successful British athletics sprinter who was suspended from competition in 2006, not because of doping, but because she missed three unannounced out-of-competition drug tests. She received a one-year ban for missing these tests, even though she was repeatedly tested at other occasions in the same period as the missed tests, without any adverse findings. This would rather indicate negligence on informing the authorities on her ‘whereabouts’ and not intentional doping-hiding behaviour. Nevertheless BOA initially imposed a lifetime ban from competing at future Olympics. Her Olympic ban was finally overturned in November 2007.

Yanina Wickmayer is a talented young tennis player from Belgium whom end 2009 was initially suspended for a year by the Flemish anti-doping authority. She had failed three times to inform about her whereabouts upon entering the Women’s Tennis Association top-50 and by consequence becoming part of the athlete pool obliged to inform about her whereabouts. She was able to defend herself in a Belgian court, pointing out shortcomings and administrative errors of the official bodies overseeing her introduction to whereabouts obligations and had the ban overturned, allowing her to play again, but her image was probably tainted forever. But her case is not over yet. Even though WADA, claiming a 2-year suspension, has decided to withdraw its appeal against the Flemish anti-doping authority following the decision of a Belgian court to invalidate the authority’s decree, the procedure between the player and the Flemish Tennis Federation has only been suspended for the moment.

Claudia Pechstein, a very successful German speed skater, was frequently tested throughout her long career, but never failed a test. On the basis of fluctuations in the number of young red blood cells in her blood (reticulocytes), she was declared guilty of blood doping in 2009 and banned from competitions for two years. This case is interesting because it was the first time that an athlete was considered guilty of doping on indirect evidence indicating the possible use of a substance that, in itself or its metabolites, were not directly identified. Perhaps the fact that Pechstein’s career started as an East German skater created a climate of suspicion because of state-organized doping practices in former East Germany. But in hindsight it is now highly likely that Pechstein’s higher than ‘normal’ levels of reticulocytes result from a genetic anomaly, and are, in her case, physiological and not the result from doping. In all there remains considerable scientific doubt on the likelihood that Pechstein did indeed use forbidden substances or methods. The case also highlights the difficulties for the CAS to rule in such complicated cases, because of the entangling of scientific, legal, economical, political and personal interests.

Pluim [5] reviewed a series of cases of doping in tennis. There were 40 cases in the 5-year period 2003– 2007, but in only 13 of these a prohibited substance was taken to enhance performance. In the other cases (68%) it was accepted at independent hearings that there was “*no intent to enhance performance*” or “*no (significant) fault or negligence*”. Recreational drugs made up 40% of the cases (11 cases of cannabis, 5 of cocaine).

Finally, an unknown number of athletes likely remain undiscovered and get away with some forms of doping. This is because of the limits to surveillance and laboratory testing technology. The ideal situation would be black and white: the forbidden substance is present or is not present in a urine or blood sample. Those two extreme cases exist, but there is, depending on the substance, often a large area of uncertainty. A test can be positive (showing the presence of a substance) when it is indeed present (true positive) or not present (false positive); conversely, a test can be negative when there is indeed no substance present (true negative) or when in fact the substance is present (false negative). Anti-doping policy enforcers need to keep false positives as low as possible, while striving for the highest sensitivity possible. The probability for false positives rises with the number of tests performed, as well as with a drop in prevalence of actual doping [6]. Furthermore, for some forms of doping practices there exist no laboratory tests. WADA does not want to publish WADA-accredited laboratories’ test performance, saying that this would permit athletes tailoring doping practice to current testing technology. At first sight, this seems reasonable, but, at the same time, it leaves room for doubt about the impartial nature of anti-doping testing. The absence of transparency is not a good gatekeeper for quality assurance.

Perversely, anti-doping is thus limited in its scope since, *in fine*, a ‘clean’ sample will never allow to fully exclude doping, while occasional sacrifice of innocent athletes from false positives appears inevitable. Even if anti-doping efforts certainly have changed current doping practices - certain types of doping cannot be used anymore because too easily discovered - the purpose of anti-doping, the celebration of ‘clean’ athletes with a strong degree of confidence, or even certainty, thus remains an unattainable objective. One is forced then to question whether the champions are ‘clean’; a question that unfortunately remains unanswered. So, if the principle of the anti-doping rule may initially seem simple, one can see that its implementation is complicated, very technical, highly costly, only partially successful and condemns athletes who did not dope or had no intent to dope.

### Enforcement of anti-doping policy today

Anti-doping is enforced by a combination of repression and surveillance. The latter includes the so-called ‘whereabouts’ rule, or the obligation for a selected pool of elite athletes to inform the anti-doping authorities where they will be each day of the year, to allow unannounced out-of (and in)-competition testing, with the obligation to be present at the announced site for one specific hour per day. The athletes have to provide this information to the authorities in advance, four times a year for three months periods at a time, using electronic and paper-based means and informing in time of any changes [4]. This rule aims at preventing out-of-competition doping in preparation for competition. To force athletes to comply, three missed tests within an 18-months period constitute a doping offence, as happened in the Ohuruogu and Wickmayer cases mentioned, and regularly occurs for other athletes. The actual testing involves providing urine samples (produced in full view by an anti-doping officer), consenting to blood sampling, and in some instances also providing hair samples for doping history and tissue for gene profiling for forensic practices. Longitudinal testing, looking for fluctuations in certain blood parameters compatible with doping, is now also being introduced. This practice, known as the ‘athlete biological passport’ (ABP), has recently led to the first indictments of athletes, based on indirect indices of presumed doping rather than laboratory tests directly showing the presence of the forbidden substances or their metabolites in urine or blood. The authorities see the ABP as an improvement of anti-doping [7]. But the ABP may produce false-positive results due to analytical variability and outlying individual patterns resulting from the effects of behaviour (training, altitude exposure) and genetics [8-10].

Mostly related to its enforcement strategies, anti-doping has a non-negligible cost [11,12]. The IOC finances half the budget of the WADA, while the other half comes from national governments. National anti-doping agencies are mostly co-financed by national sports federations and governments. Overall the tendency is towards increasing costs with a new costly anti-doping industry steadily asking for more. The application of new national anti-doping legislations also comes with an increase in cost.

Taken together, all of these costly surveillance practices seriously impinge upon the privacy of athletes and set them apart from the general population, for whom the protection of the private sphere and autonomy are generally respected in democratic societies, and are at odds with general relaxed attitudes of modern society towards human enhancement practices such as cosmetic surgery (e.g. [13], and with an increasing prevalence of the use of cognitive enhancement drugs [14].

### Other consequences of anti-doping

If anti-doping would only concern elite sport, one might accept the arguments in favour of the exceptions made in sports, for the sake of what sports aspires to be. But anti-doping has unintended side effects outside elite sports. One illustrative example of how anti-doping policies directly influence society outside the scope of competitive sport is the 2005 extension of Danish national anti-doping policy to commercial fitness clubs (gyms) in which clients engage into weight lifting and other types of exercise for health and appearance purposes, but not necessarily for sports competition [15]. Danish gyms have the obligation to put either a happy green smiley on the entrance, indicating adherence to Anti-Doping Denmark, which includes surprise urine testing of clients, or an unhappy red smiley with the explicit message that the club does not adhere to anti-doping Denmark. About 20-25% of the samples are found to contain (forbidden) anabolic steroids. As a consequence these clients are excluded from the gym in question for 2 years, and from all other gyms that adhere to the rule. According to the law the client should also be excluded from all sports in Denmark. And, as in elite sport, a refusal to be tested is counted as a positive test [15]. This example illustrates the potential for generalization of anti-doping surveillance practices in society in general. This practice is not far from the introduction of testing of students for cognitive performance enhancing substances and other drugs, and possibly other groups, like teachers, trainers, coaches, sports referees, police personnel, amateur athletes, etc. [16]. Such increased surveillance and testing would lead to increased numbers of convictions, with an important burden imposed on the judicial system and the families of the convicted. For simple reasons of stochastic and procedural error frequency, a greater number of tests would lead to a greater number of false positives, wrongly accusing innocent citizens. The prospect of such a development has worrying characteristics of a dystopia of Orwellian kind. It appears paradoxical that gym users, generally conscious about their health and complying with general preventive principles like regular exercise and a healthy diet, making a balanced decision on steroid use to aid them in attaining their aspired body form, are punished for anabolic steroid use, while the general population can freely engage in dangerous behaviour combining bad nutrition, lack of exercise, tobacco and alcohol use without much of a constraint.

### What end justifies anti-doping?

Because of the problems of current anti-doping policy the question arises as to what the reasons for the anti-doping endeavour are. The main justification for anti-doping is formulated as follows in the ‘Code’, the central document published by WADA outlining anti-doping:

"“Anti-doping programs seek to preserve what is intrinsically valuable about sport. This intrinsic value is often referred to as “the spirit of sport”, it is the essence of Olympism; it is how we play true. The spirit of sport is the celebration of the human spirit, body and mind, and is characterized by the following values: Ethics, Fair play and honesty, Health, Excellence in performance, Character and education, Fun and joy, Teamwork, Dedication and commitment, Respect for rules and laws, Respect for self and other Participants, Courage, Community and solidarity. Doping is fundamentally contrary to the spirit of sport.”^b^"

According to WADA the spirit of sport is thus the celebration of the human spirit, body and mind. The reasons advanced against doping are that it skews a level playing field, can threaten the health of the athlete, is against the spirit of sport, and incompatible with the concept of the athlete as a role model. All these arguments are problematic as the first author and several co-authors have explained before [11,17,18]. Elite sport is by definition a non-level playing field since it is about the celebration of differences. The protection of the health of the athlete argument is paternalistic and neglects the health hazards of sport itself while the distinction of avoidable and unavoidable risk is flawed. Moreover, the spirit of sport argument is fuzzy and fraught with problems, and the role-model argument is out of perspective as compared with any other role model in society. Anti-doping policies in sports have created an image of an idealized ‘perfect’ human. Obliging athletes to correspond to this ideal appears unfair compared to what is asked of other citizens.

### Why do athletes dope and will probably continue doing so?

Doping has always been part of sports. Already at its ancient Olympic roots and also at the beginning of modern sports about a century ago, athletes have always looked for and applied any ways imaginable to increase performance, be it by taking substances or by using methods [19,20]. Taking as an example the Tour de France, it can be posited that in the last century, in virtually each year the winner and/or runner-ups were either known to have used doping or strongly suspected of having done so [21]. Aspiring to organize a Tour without doping can therefore be seen as trying to invent a Tour that has never existed before. The difference between earlier and more recent Tours, admittedly important, is the kind of performance enhancing technology that has been made available by the biomedical revolution over the last decades. If in the early days of sports the arsenal of performance enhancing compounds was quite limited, today’s advances of biomedical science have indeed opened up Pandora’s box with unlimited possibilities but also increased health risks. Since a doping culture has always been part of cycling it is quite understandable that these new possibilities from bio-medical research were, and still are being exploited for performance enhancement practices by cyclists and their entourage. WADA’s claim that a culture of doping-free sport will develop and help attain the eradication of doping in sports remains to be proven; for competitive road-cycling, recent publications suggest that although doping practices certainly have changed, a culture of doping in professional cycling still prevails [22]. A provocative editorial in the journal Nature in 2007 proposed that perhaps the Tour de France should be the first competition to accept pharmacological performance enhancement [23].

Given the fact that in spite of the increasingly repressive means employed to combat doping, athletes are regularly caught - while others probably get away with it - the question arises: why do athletes continue taking the risk? The underlying question is whether current anti-doping policy in sports, striving for a world free of doping in elite sports, is commensurate with how human (doping-) behaviour is determined. Using a point of view from a sociocultural perspective, but interpreting behaviour as partly determined by our evolutionary past, we suggest that doping in athletes is perfectly natural human behaviour. Given what modern sport is today, i.e. an important entertainment enterprise in which large amounts of money go round, strongly biased towards the celebration of winners and applying the general concept of ‘winner takes all’, it is not surprising that athletes can be drawn to whatever promises an edge in competition. The official credo of the Olympic movement is ‘Citius, Altius, Fortius’, or faster, higher, stronger. It is often completed by the phrase ‘The most important thing is not to win but to take part’ [24]. But the latter is not reflected by reality. Today an important tenet of elite sport is the celebration of the winner. This puts a lot of pressure on athletes, who are perpetually seeking the competitive advantage. As Petroczi [25] states: “*using doping agents may be more of a rational, outcome optimizing behaviour than deviance*”.

What does it take to be a champion? The ingredients are a combination of talent, hard work and some luck. Talent is a licit, albeit unmerited gift and results from the genetic lottery. Hard work is essentially training and using any other means that are allowed to improve performance. Performance enhancement is a logical and essential ingredient of competitive sport. Athletes look for ways to get better, by changing their training paradigm, by eating differently, by taking vitamins, by taking licit medication, by taking supplements. A huge sports supplements industry exists and it is very common for athletes to consume a lot of substances that are not on the list of forbidden substances [26]. In 2012 the British Journal of Sports Medicine published its 28th article on the A-Z of nutritional supplements [27] and the list continues to grow. Many of the supplements do nothing, a few have an effect, but quite many may pose doping problems because of adulteration, leading to accusation of doping because of positive blood or urine samples [28] or health problems because of excess intake of some compound, erroneously seen as innocuous [29].

Modern sport puts athletes under enormous pressure to win and the use of licit substances and methods to improve performance is explicitly encouraged. The line between licit and illicit fluctuates and has dimensions that can be perceived as arbitrary.

Transgression of doping rules is not necessarily accompanied by a fundamentally different mindset as when keeping to the rules. In athletes’ minds, doping may align with illicit behaviour or with functional licit use of chemical or natural preparations [28].

### What if current zero-tolerance anti-doping policy continues?

What can be foreseen over the next decades if the central tenet of the anti-doping movement - eradication of doping - continues to drive a global agenda of surveillance and suppression of doping and doping-like behaviour? We expect that, in the short term, continued pressure from the WADA and the IOC, backed by the international UNESCO convention, will oblige an increasing number of nations to adopt specific anti-doping legislation, especially those who aspire to organize Olympic Games. There is an international tendency to combat doping and related activities like trafficking through criminal law (e.g. in Italy, France and Slovenia, see [30]), quite akin to what happened in the field of psychotropic drugs, thus criminalizing the use, possession, traffic and commerce of doping substances. This development will be accompanied by increasingly repressive measures worldwide. Increasing numbers of citizens will have to comply with compulsory drug testing for an increasingly long list of substances.

Because of such unintended effects we suggest that the debate on anti-doping should not be a matter of concern to elite-sports only. Still, anti-doping arguments are frequently formulated which explicitly or implicitly ignore the actual practical consequences of actual anti-doping policies in and outside sports (see e.g. [31,32]). We believe that this is unacceptable. Just as for the ‘war on drugs’ the consequences of the ‘war on doping’ should be fully taken into account when engaging into the debate on how to regulate the use of performance enhancing substances and methods in sports.

### The ‘war on drugs’, regulation and harm reduction

The Global Commission on Drug Policy has called the ‘war on drugs’ a failure and asks for change [33]. The alternative proposed is regulation of drug use, based on human rights and public health principles, with a combination of pragmatic policies taking into account local socio-cultural and economic specificities, and continuously adapted to on-going developments. High on the list of policies proposed are treatment and harm reduction measures. Countries like the Netherlands, Switzerland and lately Portugal, where such policies have been put in place, have shown that these strategies are accompanied by a reduction in the cost to society and the individual, by decreasing drug-related mortality and morbidity, as well as crime and its associated costs, without an increase in the prevalence of illicit drug use [33]. Examples of, mostly evidence-based, harm reduction measures include needle and syringe exchange programmes, safe use facilities, opiate substitution therapy, overdose prevention and chemical analysis of party drugs. Some of these pragmatic ways to deal with societal problems can be perceived as ‘messy’. For example, in safe injection facilities drug users bring their own supplies obtained on the black market, an idea that may be repulsive for many. But such facilities have proved their utility beyond any reasonable doubt, by reducing hepatitis and HIV transmission rates, reducing the general health burden in injecting drug user cohorts and reducing the societal costs of open drug scenes, without increasing drug use or injecting behaviour [34]. Full liberalization of all substance use seems currently politically inconceivable while harsh repression has repeatedly been shown to induce more harm to society than it prevents [33]. As a compromise between these extremes, regulation and harm reduction is a pragmatic way to dynamically deal with such behaviour [12,34]. It is dynamic because it has to be constantly adapted to changes in population behaviour.

### An alternative way to deal with doping?

The difficulties of applying a model of regulation and harm reduction to sports are of course huge, but perhaps more in line with anthropological generalizations of a socio-culturally moulded, evolutionary defined ‘human nature’. The choice between fighting doping by all means vs. regulation and harm reduction is difficult, since neither will solve the problem; no ultimate solution exists, it will remain ‘messy’. In our view, regulation and harm reduction may come with less cost to society and the individual, as compared to a zero-tolerance approach, and therefore merits to be considered. We do not have a ready-made blueprint to offer; if an easy way existed it would already have been in place. As the Global Commission on Drug Policy has proposed for the problem of illicit psychotropic drugs, we could begin thorough evaluation of the current system and accordingly begin changing it in a more reasonable way, to the benefit of the athletes and to the future of sports [33].

We would like to make some suggestions for possible strategies. To begin with, the concept of performance enhancement by means of methods or substances, including pharmacology, should be seen as a logical consequence of elite sports endeavour and not be negated by a utopic ideological ‘spirit of sport’ concept. Second, the health of elite athletes should still be protected, but taking into account the specificities of this risky profession (some sports come with a level of risk not acceptable in other professions). This can be done by continuing some form of testing, without going all the way as in today’s testing. For example, a no-starting rule for a haematocrit above a given level, however the way it got to that level, is a pragmatic way to prevent excess use of red cell mass stimulation regimes that lead to a health hazard. Sure enough, athletes will find ways to cheat a bit around such strategies, but that would be part of the game while keeping the problem within acceptable boundaries by associating such a rule with some other rules like the exclusion of plasma expanders, if warranted. The argument that it would change sports into an arena akin to Formula 1 where the best engineering team wins is only partly correct. It will still take talent, a lot of hard work and some luck to become a champion. And then, it is likely that such a scenario is already in place anyway; today well-assisted athletes may engage in complex training regimes and strategic doping while remaining undetected. Third, the list of forbidden substances can be shortened, leaving on the list only those substances with actually proven performance enhancing effects and major health hazards. For example, cannabis derivatives can be taken off the list, allowing athletes to be dealt with in the same way as the general population. The current arguments to keep cannabis on the list are flawed. There are no well-controlled trials that show any performance enhancing effect, while there is evidence for performance decreasing effects [2,3].

With regard to the general population, instead of a crackdown on steroid users in gyms and fitness clubs with compulsory testing as in Denmark [15], a harm reduction approach is probably better [12]. This has already been shown in the UK where so-called steroid clinics, giving out clean syringes and thus lowering the threshold to medical care, have led to the number of syringes handed out now outnumbering those exchanged for injection for psychotropic drugs [35]. These clinics, offering mostly free and anonymous services, make it possible to reach a previously hidden population. Potential advantages of providing harm reduction measures, besides health benefits, include the personal and direct contact with a hidden population allowing it to be informed of the risks and dangers of doping substances, and helping to take well informed decisions whether to continue use and if so in what way. These services show promise but need to be well evaluated [36]. In Switzerland the federal commission on drug-related affairs developed a conceptual model (‘the cube’) considering that for every substance, with different risk profiles, different levels of use exist (non-problematic, problematic, dependence), needing different levels of intervention at the level of prevention, treatment, harm reduction and regulation. Such a model might help to conceptualise alternative policies on performance enhancing substances respecting public health and ethical principles [37].

### What problems would arise?

As Donohue et al [38]. have noted with regard to the effect of alternative psychoactive drug policies, the two contentious questions here are also: “By how much would the prevalence and intensity of doping rise under a different regime?” and “Would reduction in other costs outweigh the risks of increased doping?”. One would have to distinguish between elite athletes, amateur athletes, minors, gym users and the public in general, since it can be expected that the answers to these questions would vary between groups. It is impossible to predict what would happen in these different groups. In elite athletes one might expect limited harm since medical supervision and health oriented testing would constrain the possibilities. In amateur athletes the possibility of refraining from sourcing substances from the black market and having access to general information and proper methods of use, might perhaps have a positive effect. It will of course be difficult to devise an optimal strategy to regulate for children specifically. Since athletic careers often start very early, the protection of young talents would be mandatory. Alternative policies should of course be continuously and extensively evaluated for desired outcomes and unintended negative consequences, carefully balancing the two.

### What are the barriers to change?

WADA has been successful in giving universal value to its Code by having the UNESCO formulate an International Convention against Doping in Sport, which has now been signed by sufficient member states of the UN (165 as of early 2012) to have universal value. The Convention’s intention is the elimination of doping in sports and it refers to WADA’s code, thus leaving it up to WADA to define doping and anti-doping. As for illicit drugs, this makes policy changes moving away from the hard line of zero-tolerance politically difficult to accept, with the added difficulty that the International Olympic Committee uses the desire of participation in the Olympic Games as a lever to force nations to implement anti-doping legislation in accordance with the WADA Code. It seems that the ideal of sport as promoted by the International Olympic Committee is attaining universal value with a legal status that tends to supersede national law. We are worried that the inertia of this system is such that in the next 10–20 years little change can be expected. We hope that in the meantime the side effects of the ‘war on doping’ will not aggravate further, and that the tendency for a fusion of the ‘war on drugs’ with the ‘war on doping’, with excessive surveillance and harsh repression of a dystopian nature will be limited before it achieves truly Orwellian dimensions.

## Conclusions

The UN Single Convention on Narcotic Drugs of 1961 and the declaration of the ‘war on drugs’ by president Nixon in 1971, were fuelled by a utopian vision of a world free of illicit psychotropic substance use - *“A drug free world—we can do it”*, (cited in [39]). Anti-doping is aiming for a similar unattainable goal, sports without doping and has adopted the —*“Just say no”*— slogan from the ‘war on drugs’ movement. The sports movement has created a utopian vision of what a human should aspire to, a young beautiful athlete with perfect behaviour. This vision is used to lever unprecedented means to combat drugs in sports and now also outside sports, that put into question all what the harm reduction movement has fought for.

Many of us would probably welcome a world without wars, drugs or doping. Still, for many reasons, perhaps partly because of our perhaps ‘innate imperfect human nature’, the daily reality is quite different. The use of licit and illicit psychoactive substances is among the leading causes of preventable death across cultures and continents. Although prevalence of illicit substance use is much lower than prevalence of licit substance abuse (e.g. alcohol, tobacco), 50 years of ‘war on drugs’ have had little effect on this prevalence but have had many negative consequences. As Room and Reuters note:

*“The system’s emphasis on criminalisation of drug use has contributed to the spread of HIV, increased imprisonment for minor offences, encouraged nation states to adopt punitive policies (including executions, extra-judicial killings, imprisonment as a form of treatment, and widespread violations of UN-recognised human rights of drug users), and impaired the collection of data on the extent of use and harm of illicit drugs, all of which have caused harm to drug users and their families”*[40].

The Global Commission and many scientists, public health and law specialists have called for a transformation of the global drug prohibition regime, with experimentation with and evaluation of alternative regulation models, and access to evidence-based drug treatment and harm reduction services for those in need. The Commission’s report asks us to

*“Break the taboo on debate and reform. The time for action is now”*[33].

The current anti-doping policy, in place for just over 10 years, resembles the ‘war on drugs’ in several aspects, as we have described. The ‘war on drugs’ and the ‘war on doping’ tend to converge, as exemplified by the categorization of steroids as class III drugs in the USA, the compulsory drug testing for Danish gym users and the presence of cannabis on the list of forbidden doping substances.

Even if sufficiently complete and accurate data on the negative aspects of anti-doping policy are still lacking, we suggest that the taboo on debate and reform also be broken in this field, now and not in 40 years. We cannot ignore the side effects of current anti-doping policy for society in general. We suggest experimentation with and evaluation of alternative ways of dealing with doping in elite (and amateur) sports, inspired by the experience gained with alternative drug policies, that are scientifically sound, respect human rights and public health, and treat athletes as ordinary humans and not as potential criminals. Regulation and harm reduction can be perceived as ‘messy’, but thanks to their pragmatic nature they might allow overall harm to society to be reduced below its current levels. The modern globalised sport entertainment industry with its almost unlimited financial means should not be allowed to hijack worldwide legal frameworks and orient society towards a zero-tolerance approach to both psychotropic and performance enhancing substances. Those who are advocating harm reduction strategies to help society live with psychotropic drugs at the lowest cost for the individual and the community should therefore be concerned about these developments. The 2012 Olympics are a good occasion to foster the debate.

## Endnotes

^a^http://news.bbc.co.uk/sport2/hi/athletics/7631774.stm, accessed May 24 2012

^b^http://www.wada-ama.org, accessed May 24, 2012

## Competing interests

No competing interests were declared.

## Disclaimer

BB and BK were under influence of a cognitive performance enhancing substance (caffeine) while preparing this paper. BK takes beetroot juice prior to trail running competitions.
